# A P5 Approach to m-Health: Design Suggestions for Advanced Mobile Health Technology

**DOI:** 10.3389/fpsyg.2018.02066

**Published:** 2018-10-31

**Authors:** Alessandra Gorini, Ketti Mazzocco, Stefano Triberti, Valeria Sebri, Lucrezia Savioni, Gabriella Pravettoni

**Affiliations:** ^1^Department of Oncology and Hemato-Oncology, Università degli Studi di Milano, Milan, Italy; ^2^Applied Research Division for Cognitive and Psychological Science, Istituto Europeo di Oncologia, Milan, Italy

**Keywords:** mHealth, technology acceptance, P5, eHealth, patient empowerment, chronic diseases

## Abstract

In recent years, technology has been developed as an important resource for health care management, especially in regard to chronic conditions. In the broad field of eHealth, mobile technology (mHealth) is increasingly used to empower patients not only in disease management but also in the achievement of positive experiences and experiential growth. mHealth tools are considered powerful because, unlike more traditional Internet-based tools, they allow patients to be continuously monitored and followed by their own mobile devices and to have continual access to resources (e.g., mobile apps or functions) supporting health care management activities. However, the literature has shown that, in many cases, such technology not accepted and/or adopted in the long term by its users. To address this issue, this article reviews the main factors influencing mHealth technology acceptance/adoption in health care. Finally, based on the main aspects emerging from the review, we propose an innovative approach to mHealth design and implementation, namely P5 mHealth. Relying on the P5 approach to medicine and health care, this approach provides design suggestions to address mHealth adoption issues already at the initial stages of development of the technologies.

## Introduction

Chronic conditions pose challenges to health systems worldwide. While acute diseases can be treated by means of *ad hoc* therapy, chronic conditions often need to be managed and contained from onset (or diagnosis) to death, with very limited possibilities of complete recovery. Such a scenario requires continuous attention and availability from the health provider as well as commitment from the patient, who must adhere to long-term therapy and change his or her lifestyle. Moreover, chronic condition management often extends the demands of care to other figures, such as patients’ caregivers, organizations, and institutions. In recent years, new technologies have emerged as an extraordinary resource to help achieve these aims (Wiecha and Pollard, 2004; Castelnuovo et al., 2015a; Gorini et al., 2016).

In the health sector, new technologies for health (eHealth) are recognized as having a great impact on health promotion and management (Bert et al., 2014; Mirkovic et al., 2014; Barello et al., 2016; Hood et al., 2016; Jacobs et al., 2016). These tools make it possible to develop and implement integrated, sustainable, patient-centered services, and promote an effective exchange between patient and doctor, with the patient taking an active role in the health care process (Samoocha et al., 2010; Barello et al., 2016). Such health technologies not only empower and facilitate the administration of continual care but also offer opportunities for maintaining patients’ active engagement in the care process by promoting patients’ psychological skills (e.g., health literacy, emotion regulation, adoption of healthy behaviors) and well-being outcomes. Eysenbach (2001) defined eHealth as a vehicle to enrich patients and stakeholders through the intersection of medical informatics and public health business. As such, eHealth promotes a new “state of mind” for medical professionals, marked by a global attitude and by the intention to improve health care locally, regionally, and worldwide.

In recent decades, eHealth has developed dramatically, consistent with the development of informatics and online technologies. One of these developments is mHealth, which refers to the implementation of *mobile technologies* as a tremendous tool to improve health outcomes (Free et al., 2013; Kumar et al., 2013; Lee et al., 2017) and to facilitate continuous health monitoring individually and from home (Stephens et al., 2017). Moreover, the global availability of mobile technologies and their ease of use have made them accessible to almost all of the population (Tokosi et al., 2017).

In recent years, mHealth obtained encouraging results in reinforcing healthy behaviors (Gurman et al., 2012; Hamine et al., 2015), mostly by using short message service (SMS) messages to improve treatment adherence; however, multiple systematic reviews still show mixed results on the effectiveness of mHealth interventions. Indeed, in spite of many studies identifying the notable advantages derived from the use of mHealth and demonstrating how these applications are appreciated by patients, acceptability, and adoption in the long term are still poor in many cases (Christensen et al., 2009; Tomlinson et al., 2013; Mohammadzadeh and Safdari, 2014; Castelnuovo et al., 2015b; Hamine et al., 2015; Guo et al., 2016; McKay et al., 2016). Also, health care apps often lack standard validation in terms of benefits, acceptance, costs, and risks (McKay et al., 2016).

Using the above-discussed evidence and controversy as a starting point, the aim of the present contribution is, after an analysis of the variables that influence mHealth technologies’ acceptance/adoption, to promote guidelines for future mHealth resources and applications.

## Issues in mHealth Adoption

There are many factors that may influence the use of mobile apps to monitor patients’ health. The first of these is related to age and expertise with technology. Chronic diseases that require life-long management generally affect elderly patients. For this reason, mHealth apps are often targeted at **middle-aged/elderly patients**, who usually have limited experience with technologies (Mattsson et al., 2017; Loerzel et al., 2018). Numerous studies reveal that older patients are less likely than younger ones to use computers regularly and, in a more general sense, have limited access to common-use technological devices (Børøsund et al., 2013). The literature on technology acceptance shows that perceived utility, perceived ease of use, and computer self-efficacy (e.g., the belief that one can use digital technology effectively) are the most important variables influencing the adoption of technology, as well as the persistence in use when difficulties are encountered (Mun and Hwang, 2003; Wu and Tsai, 2006; Kim and Chang, 2007; Wangpipatwong et al., 2008; Ward, 2013). This evidence may explain why elderly patients are less prone than younger ones to use mHealth to manage their health.

Another important factor, related to the previous one, is **usability**. Numerous applications are designed according to generic usability principles (Pagliari, 2007; Stellefson et al., 2011). However, it is possible that elderly and/or chronic patients present specific characteristics that may generate usage issues, which are difficult to predict if a “generic” user is considered as a model for usability evaluations. For example, age-related declines in sensory abilities and visual acuity may affect the ability to discriminate important information in a graphically challenging visual field (Agree et al., 2015). Regarding the content of an app, elderly patients appreciate and better understand information presented in multiple formats (e.g., when text is combined with images or videos making it easier to understand) (Bolle et al., 2016). For these usability-related reasons, older patients generally less accustomed to mobile technology than younger patients (Børøsund et al., 2013; Miller et al., 2017).

Other possible important factors related to the use of mHealth in health care are related to **patients’ preferences and their subjective, lived experience of illness**. For examples, patients may not want to have “all” the information about their disease. Instead, they may desire to know only those indications that concern them personally. For this reason, they often prefer to interact directly with health professionals, who provide them with information regarding their specific case, instead of relying on information provided by the available app (Grimsbø et al., 2012). Moreover, the mobile tool could be perceived as a “substitute” for the relationship with the clinician; in other words, patients may believe that the technology is given to them as a surrogate for the clinician, and that this tool will thus reduce their ability to interact with their doctor (Goel et al., 2011; Kondylakis et al., 2013). Such a belief, albeit erroneous, often predicts technology refusal or abandonment in the long term (Benson and Dundis, 2003; Mohr et al., 2011).

**The length of illness** and **the stage of disease** are other aspects that can determine the use of health management apps. Research performed on an interactive application (WebChoice) reveals that metastatic patients are less prone to use it compared to those who have recently been diagnosed. Metastatic patients may feel that they have already enough information about the disease and how to manage it, making them feel that the applications are not very useful to them (Grimsbø et al., 2012; Ruland et al., 2013). In contrast, immediately after diagnosis, patients tend to search for a lot of information about their disease and its treatment. Indeed, information presented during the first consultation is often forgotten or very difficult to memorize because of patients’ emotional state (e.g., anxiety and fear) (Bolle et al., 2016), so external support can be very useful to recover this information.

**Gender** is another factor that influences the use of mHealth. Men and women are equally familiar with how to use health apps through a smartphone (McKay et al., 2016); however, men are typically more confident than women regarding their ability to use technology and their experience with it (Cassidy and Eachus, 2002; Durndell and Haag, 2002). Since, as noted above, computer self-efficacy is an important variable influencing technology acceptance, gender may also indirectly affect mHealth adoption.

Finally, **psychological variables** such as cognitive representation of the disease, distress, and anxiety may significantly influence the adoption of mHealth (Ruland et al., 2013; Beiwinkel et al., 2017). For example, having low self-control and self-efficacy could reduce patients’ confidence in their ability to deal with their symptoms (Gysels and Higginson, 2007). Furthermore, in some situations, interacting with mHealth apps creates a fear of “getting even more problems” (Kessel et al., 2017). Indeed, some health-related apps ask users to comply with novel requests for health management. For instance, patients may have to monitor their smartphones to respond to daily messages asking them to report information on a web-based platform (e.g., glycemic values in diabetes), or they may be asked to use various app functions frequently (e.g., playing with an educational serious game and filling in online questionnaires on their health status). If the patient is not convinced about the utility of these tools for his/her health management, such new commitments may be a source of further stress and, ultimately, of negative attitudes towards the treatment. In such situations, patients may not only abandon the mHealth tools but also lose faith in their health providers, resulting in detrimental effects on the effectiveness of the health management process as a whole. Also, patients do not always have good insight into their health conditions; as such, they may wrongly think that they do not need any kind of support from mHealth (Nijland et al., 2011), causing them to demonstrate active and voluntary resistance to any kind of proposed tools.

This review of the literature sheds some light on important demographic, user-experience-related, and psychological factors that may have an impact on mHealth acceptance by patients involved in interventions. It is certainly difficult to address all of these factors in any possible intervention involving mobile technology; however, a specific theoretical perspective, rooted in an approach to medicine and care, could be useful not only to adapt already-designed devices and applications but also to develop future mHealth tools. On the one side, these advanced resources would make good use of all the opportunities offered by mobile technologies; on the other side, they would be designed according to general principles allowing health professionals to consider in advance (and possibly avoid) the acceptance issues highlighted above.

## A P5 mHealth Approach

**FIGURE 1:**
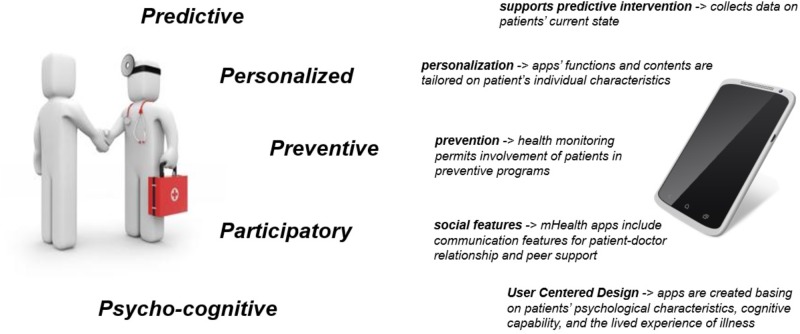
The P5 approach to medicine (left) and its design suggestions for mHealth development.

Some years ago, a system approach called “P4 medicine” was proposed (Hood and Friend, 2011; Hood and Flores, 2012). This approach was intended as a sophisticated extension of what is usually called “personalized medicine.” Specifically, the four Ps referred to the Predictive, Personalized, Preventive, and Participatory aspects of clinical medicine (Price et al., 2009; Auffray et al., 2010). A few years later, a P5 medicine approach was proposed (Gorini and Pravettoni, 2011; Pravettoni and Gorini, 2011), where the fifth P referred to the Psycho-cognitive aspects that play a significant and unique role in the way in which an individual experiences emotional events, copes with illness, and makes decisions about his/her own health. The time is ripe to use the P5 approach in combination with the most recent advances in technology in order to challenge the health care and technology industries to find innovative and personalized ways to improve the overall quality of care. In particular, a P5 mHealth approach can be developed (see Figure 1).

Such an approach may lead to a new generation of mHealth apps based on the features reflecting the original P5 construct. Specifically, mHealth apps should be as follows:

### Predictive

Collecting data on the patient’s current health state without the need for frequent physical encounters with the health care provider (physician, psychologist, nurse, etc.) will increase the amount of available information, allowing for a more precise prediction of the patient’s future health state. Specifically, mHealth apps can collect data by means of various peripherals and in-app tools. For example, they can collect physiological parameters through integration with wearable technologies (e.g., heart rate variability and skin conductance), or they can store user-generated information related to medical values (e.g., glycemic level in diabetes) (Fontecha et al., 2015; Os et al., 2017). Moreover, they can include *ad hoc* or validated questionnaires to be filled in by health providers, caregivers, and patients (e.g., data related to the patient’s functional or psychological status) (Gorini et al., 2015; Renzi et al., 2017). In most cases, such data are actually used for analyses to be included in scientific publications, but their utility for therapy and health management could be further exploited. Indeed, based on predictive models, mHealth tools of the future can provide specific information from autonomous data-analysis, in order to help both the physician and the patient to foresee the patient’s future health state, management issues, and possible modifications to the patient’s therapy regimen and/or health management activities, as well as interventions targeted to different aspects of the patient’s well-being (e.g., improving relaxation and positive emotions or promoting engagement in self-actualizing experiences).

Examples: Chih et al. (2011) used a mobile app with patients addicted to alcohol; implementing a Bayesian predictive model, they were able to predict the likelihood of relapse based on repeated self-report questionnaires investigating relapse history and psychological aspects related to the recovery progress. Wearable and mobile technology (e.g., actigraphy devices) have been used to satisfactorily predict factors relevant for quality of life, such as sleep efficiency based on physical activity during waking hours (Sathyanarayana et al., 2016).

### Personalized

Apps’ functions and contents (including requested information, feedback to the patients, etc.) should be tailored to the patient’s individual bio-psycho-social characteristics to provide more useful, more accepted by patients, and non-redundant information (Gorini et al., 2015; Pravettoni et al., 2016). Moreover, the personalization factor also relates to the possibility for the patient to express him- or herself through the use of the application. For example, app functions and automatic communications will be not generalized to patient populations but rather will be based on individual characteristics. mHealth tools of the future should be able to adapt their functioning and interfaces to previously collected data on each person’s specific features (e.g., age, sex, life cycle phase, and temporary health state, as well as attitudes, needs, and preferences). It is essential not to ask patients to perform tasks/activities that are difficult or even dangerous for them to achieve (e.g., asking patients with a cardiac illness to perform too much physical activity). Moreover, properties of applications devoted to user engagement (e.g., gamification aspects) should not be underestimated; for example, serious games have been developed with avatars designed for the user to see examples of healthy activities and be more motivated to replicate them in real life (Proteus effect) (Yee and Bailenson, 2007; Murray et al., 2013; Villani et al., 2018); such tools are also interesting resources that can be used to guarantee personalization features to users.

Examples: In the medical field, innovative approaches have been proposed to use avatars (or, better, “supermodels” as this is the term used) of the patients to include key medical components of the patient, as well as predictive analytics, so as to tailor health interventions to the individual (Brown, 2015); these could be updated with user-generated characteristics to aid in diagnosis and treatment with self-reported data (Triberti and Chirico, 2016).

### Preventive

A long-term monitoring of patients’ health would make it possible to provide timely preventive interventions and improved patient involvement in preventive programs. As noted previously, such technologies present remarkable opportunities in terms of data collection and analysis, which should be further exploited in terms of aid to health care management and therapy effectiveness. Collected data may be the basis for preventive interventions, in order to modify users’ behavior and responses before problematic consequences arise. For example, engaging health apps may not only address existing problems but also help to positively change users’ behavior, attitudes, and motivation toward health care management and treatment adherence. In this sense, mHealth should not be used as a digital assistant to treatment (i.e., “virtual medicine”) but rather as an empowerment technology that directly influences patients’ everyday activities in order to promote a healthier lifestyle and prevent negative consequences of illness.

Examples: mHealth apps for prevention have been found to be effective and positively accepted by patients. For example, some of these apps make use of automated texting to reinforce healthy behavior (e.g., physical activity) or boost it when the patient is reluctant to perform it (Martin et al., 2015).

### Participatory

Recent approaches to medicine highlight that the most successful interventions are those that recognize patients not as passive recipients of care but rather as active decision makers who can make use of their own social support resources (McNutt, 2004; Lucchiari et al., 2010; Cutica et al., 2014). Patient–doctor communication is fundamental in any health management process; in this sense, mobile-based technologies should not be used as a substitute for this relationship, but rather specific functions intended to promote it should be envisaged by designers and policy makers. These include Instant Messaging functions and social networking features, as well as the possibility for the patient to have a personal profile that is continually updated with the patient’s personal information. Second, peer support has been recognized as a tremendous opportunity for positive and effective health management (Fisher et al., 2012; Merolli et al., 2013): patients benefit from interaction and collaboration with other patients who are living with similar experiences and could give them useful suggestions, as well as simply sharing their experiences, in order to empower one another’s health management abilities. mHealth tools should include such opportunities by making use of social/interpersonal technologies embedded in mobile interfaces.

Examples: An integrative review (McColl et al., 2014) found that peer support (traditionally via telephone, then via mobile functions such as texting) increases engagement in wellness activities, reduces depressive symptoms, and improves social support-related coping. Studies show that, when mobile apps include features for communication with the clinician, patients use them to transmit not only medical information but also personal needs and feelings (Triberti et al., 2018).

### Psycho-Cognitive

Using a user-centered design approach, apps are created on the basis of patients’ psychological characteristics, their cognitive capabilities, and their lived experience of illness. Indeed, research on health technology shows that eHealth may systematically fail when the patient’s subjective experience has not been taken into consideration from the first steps of the technology design (Triberti and Barello, 2016). These features will improve the patients’ abilities to manage their emotions, to cope with their illness, and to make decisions about their health, becoming active actors in the health management process. In other words, the design and development of advanced mHealth tools not only should make use of the tremendous opportunities offered by these tools (e.g., continual monitoring, reaching the patient wherever he/she is, integration with multiple devices, and functions by means of apps) but also should be based on the application of specific research techniques in order to (1) identify users’ characteristics, needs, and contexts of use; (2) develop efficient and personalized decision support tools to help patients to make the right decisions about their health; and (3) test the technology’s effectiveness and adequacy at multiple steps of their implementation in the field.

Examples: An effective self-management platform could be based on research on patients’ needs and cognitive style, by using multiple design-oriented methods (Pravettoni et al., 2015; Kondylakis et al., 2017). Regarding psycho-cognitive aspects to be included in implementation, Kondylakis et al. (2014) developed and validated ALGA-C, a web-based tool featuring a questionnaire for cancer patients (analyzing psycho-cognitive aspects ranging from personal needs to cognitive/decision making style) and a profiling mechanism. This tool enables the clinician to modulate language, communication style, and content of the subsequent encounters with the patient, in order to empower mutual understanding and collaboration. This tool is an example of automated data-gathering tools that could be adapted to mHealth technology in order to adapt the intervention to the uniqueness of the patient over time, taking into account not only his/her physical health status but also psychological processes influencing adoption.

## Conclusion

Despite their increasing popularity, the literature shows that there are still significant limitations in the acceptance and long-term adoption of mHealth apps, mostly because these technologies do not take users’ needs and contexts of use into account from the first steps of design and implementation. Here, we presented a “P5 mHealth approach” as a set of suggestions (and related examples) for aspects of the mobile technologies that could be exploited in the future advanced mHealth resources (see Table 1). Rather than relying on the intrinsic properties of technologies only, health management processes should appreciate the uniqueness of patients in order to foster mHealth abilities in terms of prediction, personalization, prevention, participatory features, and the psycho-cognitive uniqueness of the individual. Future studies in the field would implement such suggestions in design, as well as to test their utility for identifying possible improvements for the already-existing mHealth tools that are hindered by adoption issues.

**Table 1:** Design suggestions for mHealth applications from the P5 perspective.

5 Ps	Definition	How to achieve It in Design
*Predictive*	Collecting data on the patient’s current health state to increase the amount of available information, allowing for a more precise prediction of the patient’s future health state.	– Collect physiological parameters through integration with wearable technologies; – Store user-generated information related to medical values; – Include *ad hoc* or validated questionnaires to be filled in by health providers, caregivers and patients.
*Personalized*	Tailoring apps’ functions and contents on the patient’s individual bio-psycho-social characteristics to provide more useful and non-redundant information.	– Adapt mHealth’s functioning and interfaces to previously collected data on each person’s specific features; – Design application features devoted to user engagement.
*Preventive*	Long-term monitoring of patients’ health to provide timely preventive interventions and increased involvement of the patient in preventive programs.	– Collect data in order to modify users’ behavior and responses before problematic consequences actually show up; – Configure an empowerment technology that directly influences patients’ everyday activities in order to promote a healthier lifestyle.
*Participatory*	Recognizing patients not as passive recipients of care but rather as active decision makers who can make use of their own social support resources.	– Sustain patient–doctor communication, as well as communication with designers and policy makers (Instant Messaging functions, social networking features); – Promote the possibility for the patient to have a personal profile that is continually updated with the patient’s personal information; – Make use of social/interpersonal technologies embedded in mobile interfaces in order to empower health management abilities via peer support.
*Psycho-cognitive*	Improving the patients’ ability to manage their emotions, to cope with their illness and to make decisions about their health, becoming active actors in the health management process.	– The design and development of advanced mHealth tools based on the application of specific research techniques; – Identify users’ characteristics, needs and contexts of use; – Develop efficient and personalized decision support tools; – Test technology’s effectiveness and adequacy at multiple steps of its implementation in the field.


## Author Contributions

AG conceived the ideas presented in the article and wrote the first draft. KM and ST contributed with discussion on the ideas presented in the article and edited the manuscript. VS and LS performed relevant bibliographic search and contributed to revisions. GP supervised the whole process and contributed with important intellectual content.

## Conflict of Interest Statement

The reviewer SS declared a past co-authorship with one of the authors ST to the handling Editor. The authors declare that the research was conducted in the absence of any commercial or financial relationships that could be construed as a potential conflict of interest.
